# Mapping and Validation of QTLs for the Amino Acid and Total Protein Content in Brown Rice

**DOI:** 10.3389/fgene.2020.00240

**Published:** 2020-03-17

**Authors:** Su Jang, Jae-Hyuk Han, Yoon Kyung Lee, Na-Hyun Shin, Yang Jae Kang, Chang-Kug Kim, Joong Hyoun Chin

**Affiliations:** ^1^Department of Plant Science, Plant Genomics and Breeding Institute, Research Institute of Agriculture and Life Sciences, Seoul National University, Seoul, South Korea; ^2^Department of Integrative Bio-Industrial Engineering, Sejong University, Seoul, South Korea; ^3^Division of Applied Life Science (BK21 Plus Program), Plant Molecular Biology and Biotechnology Research Center, Gyeongsang National University, Jinju, South Korea; ^4^Division of Life Science, Gyeongsang National University, Jinju, South Korea; ^5^Genomics Division, National Institute of Agricultural Sciences, Jeonju, South Korea

**Keywords:** rice (*Oryza sativa* L.), amino acid content, protein content, quantitative trait loci, brown rice, genotyping-by-sequencing

## Abstract

Highly nutritious rice production will be benefited with the improvement of amino acid content (AAC) and protein content (PC). The identification of quantitative trait loci (QTLs) associated with the PC and AAC of rice grains could provide a basis for improving the nutritional value of rice grains. Here, we conducted QTL analyses using recombinant inbred lines from the cross between *indica* (Milyang 23 or M23) and *japonica* (Tong 88-7 or T887) rice varieties, afterward employing genotyping-by-sequencing to obtain a high-density genetic map. A total of 17 and 3 QTLs were detected for AAC and PC, respectively. Among them, two QTLs associated with more than 10 AACs, *qAAC6.1* and *qAAC7.1*, were identified for the first time in this study. Each favorable allele that increased the AAC of the two QTLs was derived from M23 and T887, respectively. Allelic combination of *qAAC6.1*^*M*23^ and *qAAC7.1^T887^* showed significantly higher content of associated amino acids (AAs) than other allelic combinations. Near-isogenic line (NIL) possessing *qAAC7.1^T887^* with M23 genetic background had significantly higher AACs than both parents. These results indicate that the pyramiding of QTLs would be useful in developing brown rice with a high AA and protein content.

## Introduction

Rice has been a staple food for thousands of years, especially for Asians and Africans. The global consumption of rice is gradually increasing as its dietary benefits in human nutrition has been more factually accepted in many countries. In most rice growing countries, rice eating qualities have evolved through highly polished rice by utilizing advanced milling technologies. In the process, rice bran, which is the most nutritional component of rice, is easily removed. Common polished rice loses various phytochemicals, vitamins, and minerals (Vetha-Varshini et al., 2013). In healthy rice breeding programs, the promotion for brown rice consumption can be a powerful option. Furthermore, brown rice can be a better option for agriculture policy makers and economists to secure higher rice production.

The protein content (PC) and amino acid content (AAC) may not be *indica–japonica* specific traits. The positive effects of increasing PC and AAC could be derived either from *japonica* or from *indica*. Although the identification of the associated quantitative trait loci (QTLs)/genes for AAC is important, there are very few studies on the trait identification on AAC. Wang et al. (2007) reported the QTLs for AAC in milled rice, using recombinant inbred lines (RILs) from a cross between Zhenshan 97 (*indica*) and Nanyangzhan (*japonica*). In all amino acids (AAs), the AAC of Zhenshan 97 was larger than that of Nanyangzhan in the 2-year experiments. In the study, several QTL clusters for AAC were identified on chromosomes 1, 2, 3, 4, 7, 8, and 10. There were some large effect QTLs for Asp, Thr, Gly, and Ala on RM472–RM104 on chromosome 1 (phenotypic variance explained, PVE = 17.5–33.3%), and Gly, Arg, Met, and Pro on RM125–RM214 on chromosome 7 (PVE = 4.68–7.69%). Another study on the QTL identification for AAC in brown rice was reported (Yoo, 2017). In the study, the QTL for AAC including Ala, Val, Leu, Ile, and Phe was the only QTL cluster on chromosome 3 in Dasanbyeo (*indica*) and TR22183 (*japonica*) RILs. In the same study, another QTL for Lys was identified on chromosome 3. In both the studies, the precise linkage mapping analysis was limited due to lack of markers in the QTL regions.

On the other hand, several QTLs for PC were identified. The rice PC varied from 4.9–19.3% in *indica* and 5.9–16.5% in *japonica* (Lin et al., 1993). The earliest study on QTL mapping for PC was conducted using Zhenshan 97/Minghui 63 RILs (Tan et al., 2001). Two QTLs on chromosomes 6 and 7 were identified using the RFLP marker system. The *Wx* marker associated with amylose content (AC) was linked to QTLs on chromosome 6. The QTL for PC linked to ACs were identified repeatedly in the following studies. The QTL near *Wx* (RM190) on chromosome 6 explained 19.3% PVE in the other study (Yu et al., 2009). Two major QTLs for PC were identified on chromosomes 6 (RM588–RM540) and 7 (RM5436–RM6776) (Lou et al., 2009). Eight QTLs for PC included two QTLs on chromosome 6 (Kinoshita et al., 2017) and a new QTL for PC and AC on chromosome 8 was reported.

Due to the complex genetic structure in the study of AAC and PC, very few genes were cloned and functionally investigated. A major QTL for grain PC (GPC), *qPC1* (a putative AA polymerase, *OsAAP6*) on chromosome 1, was cloned and characterized (Peng et al., 2014). *qPC1* is a QTL cluster exhibiting the highest PVE (32.4%), conferred by the Zhenshan 97 allele.

Currently, the genes/QTLs for protein turnover and complicated AA catabolism have not been characterized well – especially in brown rice. The linked QTLs for AA should be mapped within narrow regions on the chromosomes, and some candidate genes should be suggested for future studies.

In this study, other *indica–japonica* RILs have been analyzed for AAC and PC in brown rice. Followed by the identification of thousands of single nucleotide polymorphic loci (SNPs) using the genotyping-by-sequencing (GBS) technique, several QTLs were identified in high-resolution genetic maps. Furthermore, validation of major QTL, *qAAC7.1*, were conducted using backcross progeny. The QTLs identified in this study and their closely linked markers could be used to develop high nutritional rice breeding program.

## Materials and Methods

### Plant Materials

The F_15_ RILs, derived from a cross between Milyang 23 (M23, *indica*) and Tong88-7 (T887, *japonica*), were developed at Seoul National University in Korea (Jiang et al., 2008). The plant materials, together with the parental lines, were grown at the Seoul National University Experimental Farm in Suwon. The plants were transplanted to one seedling per hill at a planting density of 30 × 15 cm. Plants were cultivated under normal fertilizer conditions (N–P_2_O_5_–K_2_O = 100–80–80 kg/ha). The rice field was regularly irrigated.

### Quantification of Amino Acid Content and Total Crude Protein Content

Unhulled rice grains for assay were harvested at 50 days after heading, and were naturally dried on drying beds with transparent roofs for 14 days. Unhulled grains were stored at 10°C, until all samples for assay were ready.

Dehulling and grinding were conducted simultaneously for all samples. Before grinding, the brown rice grain was cleaned by removing broken, green, and immature grain. A total 30 g of brown rice grain was ground at a time and the aliquots of powder were used to each assay.

AAC was determined using the high-performance liquid chromatography (HPLC) method. One hundred milligrams of powdered milled brown rice samples were collected using more than 100 seed samples of different plants of each RIL. Afterward, 40 mL of 6 mol/L HCl was added, and hydrolysis was conducted for 24 h at 110°C. Samples were analyzed by HPLC (Dionex Ultimate 3000, Thermo Fisher Scientific) after pre-injection derivatization (Henderson et al., 2000). The primary AAs were reacted first with *o*-phthalaldehyde (OPA) and the secondary AAs were derivatized with fluorenylmethyl chloroformate (FMOC) before injection. The contents of each AA in each hydrolysate were calculated in reference to the standard AA and expressed as mg/g rice powder. The levels of the 16 AAC traits of rice grains (Ala, alanine; Arg, arginine; Asx, aspartic acid + asparagine; Glx, glutamic acid + glutamine; Gly, glycine; Ile, isoleucine; Leu, leucine; Lys, lysine; Met, methionine; Pro, proline; Phe, phenylalanine; Val, valine; Tyr, tyrosine; His, histidine; Thr, threonine; Ser, Serine) were obtained. The total nitrogen of crude protein was determined by using the micro-Kjeldahl method. The PC was calculated using a conversion factor of 5.95 (Jones, 1931).

### Genotyping-by-Sequencing Library Preparation and Genotyping Analysis

The whole genomic DNA for each RIL and parents were isolated from 1-month-old leaf tissues using the CTAB extraction method (Murray and Thompson, 1980). The quality and quantity of DNA were measured using PicoGreen (Invitrogen, Carlsbad, CA, United States), before diluting it to a concentration of 10 ng/μL for GBS. A GBS library was prepared with the restricted enzyme *Ape*KI, as described by Elshire et al. (2011). The quantity and quality of the GBS library was determined through the Bioanalyzer Kit (Agilent Genomics, Santa Clara, CA, United States). For accurate variation calling from our RIL population of inter subspecific cross, we prepared subset of rice reference genomes with *Ape*KI restriction sites, which are target genomic regions for GBS genotyping with IRGSP 1.0 pseudomolecule. We *in silico*-searched the *Ape*KI sites in the reference genome sequences, and split it into fragments. The subset of genome fragments smaller than 2 kb were collected. The genome subsets were indexed and the GBS short reads were mapped using BWA software (Li and Durbin, 2009). The genotypes of the RIL population were determined into homo- and heterozygous genotypes by the software SAMtools, with default parameters. For cross-validation, the parents, genomic DNA of M23 and T887, were fully sequenced using the HiSeq 2000 platform (Illumina, Inc., San Diego, CA, United States) to compare allele contents for each locus with GBS results. Only the called SNPs from the GBS results which matched with resequencing results of the parents, M23 and T887, were chosen for the following analyses.

### QTL Mapping and Validation

Genetic map construction and QTL analyses were carried out using the software ICIMapping 4.1 (Meng et al., 2015). All 26,424 SNPs discovered through GBS were binned using the BIN function allowing only SNPs which have less than 10% missing rate of individuals. A total of 1,327 bin markers were used to construct the linkage map, and the ordering of the markers within each chromosome was based on the physical position of the Nipponbare reference genome, IRGSP 1.0 (Sakai et al., 2013). The recombination distance was calculated using the Kosambi mapping function (Kosambi, 1943). An inclusive composite interval mapping (ICIM) method was used to detect additive QTLs. The logarithm of odds (LOD) values greater than 2.5 was used to declare significant QTLs. Backcross population for selecting *qAAC7.1* recombinants was derived from BC_3_F_1_ plants with *qAAC6.1* allele of recurrent parents (M23) and heterozygous *qAAC7.1*. *qAAC6.1* allele were discriminated using two insertion/deletion (InDel) markers, id3428419 and id4203043. Primers used in recombinants selection were listed in Supplementary Table S1. Near-isogenic line (NIL) was developed by repeated backcrossing with the M23 as recurrent parent. The backcross progeny was selected by marker-assisted foreground selection using two InDel markers, id4638739 and id5227020 (Supplementary Table S1). PCR amplification was performed in 20 μL reaction mixture containing 100 ng of DNA, 0.5 U Prime Taq polymerase (GeNet Bio, South Korea), 1× PCR buffer, 0.5 μM of each primer, 0.5 mM of dNTPs. PCR condition was 94°C for 5 min, followed by 30 cycles of 94°C for 30 s, 58°C for 30 s, 72°C for 30 s, and final extension at 72°C for 10 min. A total 176 SNP type assays (Fluidigm, San Francisco, CA, United States) were employed to select genetic background of NIL. All assays were designed based on polymorphism between *indica* and *japonica* (unpublished). All statistical analyses were performed using R version 3.4.3.

## Results

### Phenotypic Analysis

**FIGURE 1:**
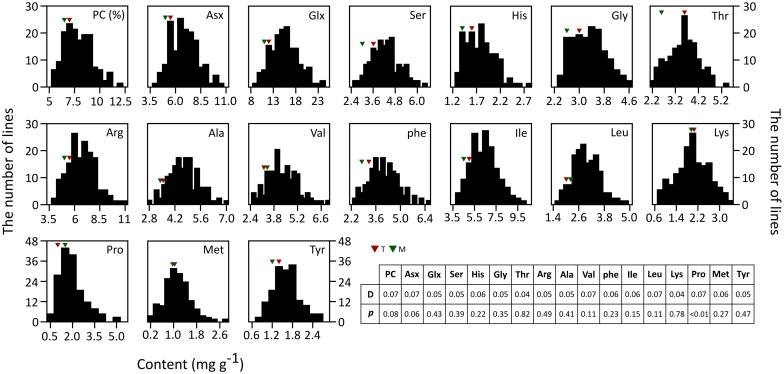
Phenotypic distributions of 16 AACs and PC in brown rice of 155 RILs. Each AAC and PC is represented in percentage and mg/g, respectively. The result of Kolmogorov–Smirnov test used to test normality of distribution is shown in table (*p* ≤ 0.05).

The 16 AACs and PC of brown rice in both parents and RILs were analyzed (Figure 1). Unlike the other AAs, the values of Met, Val, Leu, Lys, and Pro were higher in T887 than in M23. The phenotypic variation of total PC and most AACs showed a continuous and normal distribution in RILs, based on the Kolmogorov–Smirnov normality test. However, Pro content were asymmetrically distributed with a long tail to higher values, showing a positive skewness (*D* = 0.1, *p* < 0.01). Transgressive segregations in both directions were observed for PC and all AAC traits in the RIL population showed values higher or lower than that of both parents. Significant positive correlations were found between PC and most AACs, except for Pro (Supplementary Figure S1). However, positive correlation between PC and two AAs, Lys and Pro, were relatively weak (correlation coefficient <0.2). Each AAC was significantly positively correlated with the other AAC traits.

### GBS Analysis and Genetic Map Construction

A total of 2,468,603 and 256,571 variants were found in M23 and T887 genome sequence, compared to Nipponbare reference genome (IRGSP 1.0), respectively. Only the called SNPs from the GBS results of RILs which matched with resequencing results of the parents were chosen for the following analyses. A total of 26,424 SNPs were called successfully from the GBS result. All the SNPs showing the same allele calls across the lines were filtered and binned. Consequently, 1,327 SNPs were regarded as the representative markers of each linkage block in the population (Supplementary Figure S2). The SNPs identified from GBS were non-uniformly distributed, resulting in large gaps across all chromosomes, especially in chromosome 4 (66.1–111.34 cM), chromosome 6 (3–26 cM), chromosome 8 (7.5–38.5 cM), and chromosome 12 (12.9–36.3 cM and 50.7–77.2 cM). The total length of the genetic map was 1,421.6 cM. The average physical distance between markers were 3.56 Mb.

### QTL Analysis for PC and AACs

**FIGURE 2:**
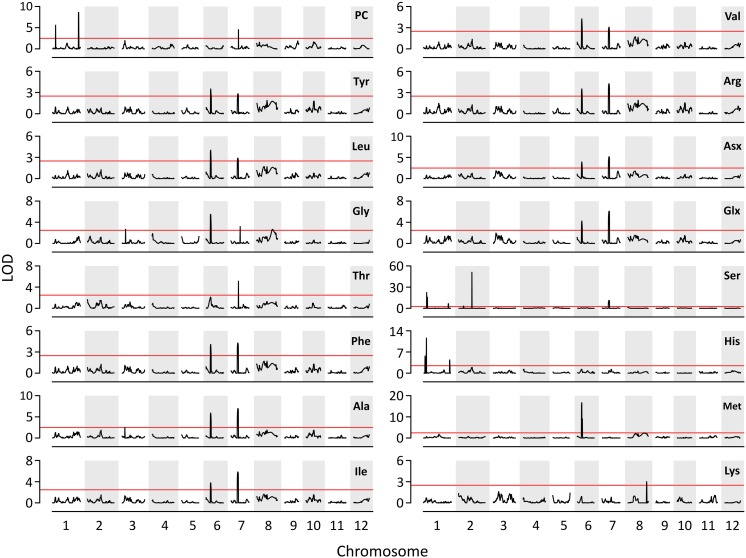
The results of QTL analysis for AAC and PC. Red horizontal line indicates LOD threshold (>2.5).

A total of three QTLs for PC and 17 QTLs for AACs were identified on six chromosomes: 1, 2, 3, 6, 7, and 8 (Figure 2). Three QTLs for PC, including *qPC1.1*, *qPC1.2*, and *qPC7.1*, were co-located with QTLs for Ser (*qAAC1.3*), His (*qAAC1.6*) and Thr (*qAAC7.2*), respectively. The positive QTL effects of *qPC1.1* and *qPC7.1* were derived from T887 (Supplementary Table S2), whereas the positive effects of *qPC1.2* derived from M23. Among them, *qPC1.2* had the largest additive effect, explaining 18.1% of the phenotypic variance in PC.

Except for proline, the QTLs for all the other AAs were identified in this study (Figure 2). Five QTLs (*qAAC6.1*, *qAAC6.2*, *qAAC7.1*, *qAAC7.2*, and *qAAC8.2*) were linked with the essential AAC. *qAAC2.2* for Ser showed the highest LOD score greater than 51.7, explaining 29.5% of phenotypic variance (Supplementary Table S2). QTLs for Ser were the most frequently detected in RILs, with a total of six loci including *qAAC1.3*, *qAAC1.4*, *qAAC1.5*, *qAAC2.1*, *qAAC2.2*, and *qAAC7.1*.

**FIGURE 3:**
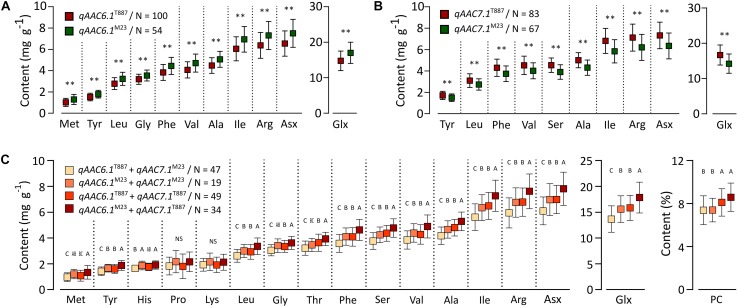
Allelic effects for AACs in 155 RILs. Allelic effects of *qAAC6.1* **(A)** and *qAAC7.1* **(B)**. Asterisk represents statistically significant difference (**p ≤ 0.01). Red box and green box represent the average AAC of each line containing the alleles originated from T887 and M23, respectively. **(C)** Effect of different allelic combination of *qAAC6.1* and *qAAC7.1*. Different letters indicate significant differences between groups (*p* ≤ 0.05, Tukey’s HSD test). N, the number of lines.

Two major QTLs (*qAAC6.1* and *qAAC7.1*) affected several AACs simultaneously. *qAAC6.1* affected a total of 11 AACs (Ala, Arg, Asx, Glx, Gly, Ile, Leu, Met, Phe, Tyr, and Val), explaining 7.8–13.8% phenotypic variance (Figure 2 and Supplementary Table S2). *qAAC7.1* had an effect to 10 AAC traits, showing 3.2–16.3% PVE. This QTL included two LOD peaks. There was one LOD peak for Arg, Ile, Leu, and Phe, detected between 4.86 and 5.02 Mb, and another LOD peak for Ala, Asx, Glx, Ser, Tyr, and Val, detected in the 5.02–5.21 Mb interval. Since each LOD peak area overlapped at around 5.02 Mb, the two detected loci were designated as one QTL, *qAAC7.1*. The lines carrying the *qAAC6.1* allele derived from M23 (*qAAC6.1*^*M*23^) showed significantly higher contents of 11 AAs than lines with *qAAC6.1*^T887^ (Figure 3A), while lines with *qAAC7.1*^T887^ showed significantly higher contents of 10 AAs than lines with *qAAC7.1*^*M*23^ (Figure 3B). Allelic combinations of two additive QTLs, *qAAC6.1* and *qAAC7.1*, additively affected most AACs, showing no epistatic interaction (Figure 3C). Notably, the combination of *qAAC6.1*^*M*23^ and *qAAC7.1*^T887^ exhibited significantly higher contents of 11 AAs, including Tyr, Leu, Thy, Phe, Ser, Val, Ala, Ile, Arg, Asx, and Glx than the allelic combinations of both parents (*qAAC6.1*^*M*23^/*qAAC7.1*^*M*23^ and *qAAC6.1*^T887^/*qAAC7.1*^T887^).

### Validation of qAAC7.1

**FIGURE 4:**
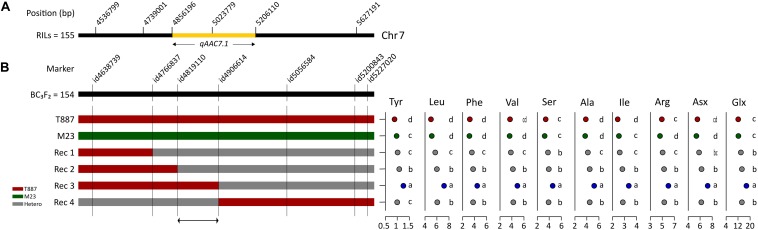
Selection of *qAAC7.1* recombinants. **(A)** *qAAC7.1* region detected by primary mapping in RILs. **(B)** Selection of *qAAC7.1* recombinants using BC_3_F_2_ population. Different letters indicate significant differences between groups (*p* ≤ 0.05, Tukey’s HSD test). Red and green bar indicate the homozygotes with T887 and M23 allele, respectively. The gray bar indicates the heterozygotes.

**FIGURE 5:**
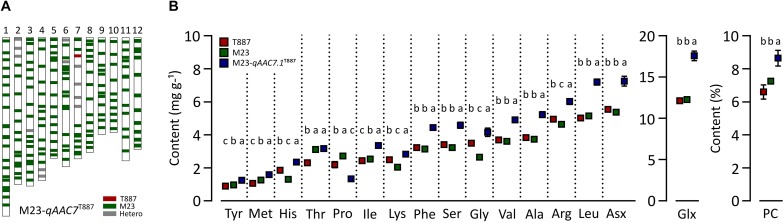
Validation of *qAAC7.1* effect. **(A)** Genetic background of M23-*qAAC7.1*^T887^. Red and green bar indicate the homozygous alleles from T887 and M23 allele, respectively. The gray bars indicate the heterozygous allele. **(B)** Allelic effect of *qAAC7.1^T887^* for AACs and PC. Different letters indicate significant differences between groups (*p* ≤ 0.05, Tukey’s HSD test).

In order to confirm genetic effect of *qAAC7.1* detected in primary mapping (Figure 4A), 154 BC_3_F_2_ populations, which were derived from BC_3_F_1_ individual plant with *qAAC6.1* allele of recurrent parents (M23) and heterozygous *qAAC7.1* in the genetic background of M23, were used. Five recombinant plants were selected with two InDel markers, id4638739 and id5227020 (Figure 4B). Five additional markers were used to narrow down target region and, consequently, a recombinant plant with homozygous *qAAC7.1^T887^* between id4819110 and id4906614 markers was selected (Figure 4B). NIL carrying homozygous *qAAC7.1^T887^* with M23 genetic background (M23-*qAAC7.1*^T887^) had genomic composition with recovery of recurrent genotype at 163 out of 176 loci (Figure 5A). M23-*qAAC7.1*^T887^ possessed a combination of *qAAC6.1*^*M*23^ and *qAAC7.1*^T887^, which showed higher AACs than the other allelic combinations in RILs. As expected, this line had significantly higher contents of *qAAC7.1*-related AAs than both parents. In addition, M23-*qAAC7.1*^T887^ had also increased PC and the others AACs, including Met, His, Lys, and Gly than both parents (Figure 5B).

### Comparison of QTLs for PC and AACs in the Present and Previous Studies

**FIGURE 6:**
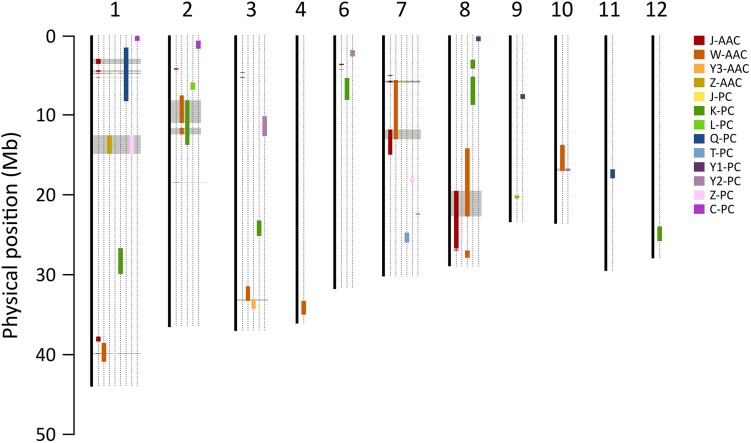
Comparison of QTLs for PC and AAC in the present and previous studies. Color bars on chromosomes represent QTL regions for AAC and PC reported by the present and previous studies. Gray blocks indicate the loci where two more QTLs were co-located. Each prefix of trait indicates the previous study (J, this study; C, Chattopadhyay et al., 2019; K, Kinoshita et al., 2017; L, Lou et al., 2009; Q, Qin et al., 2009; T, Tan et al., 2001; W, Wang et al., 2007; Y1, Yun et al., 2014; Y2, Yu et al., 2009; Y3, Yoo, 2017; Z, Zhong et al., 2011).

The consensus map of QTLs between present and previous studies were constructed to compare the loci where several QTLs were co-located. Marker positions of previous studies were determined using the Nipponbare reference genome (IRGSP 1.0). A total of five QTL clusters, on chromosome 1, 2, 7, and 8, were found to overlap the previously reported QTLs (Figure 6 and Supplementary Table S3). In particular, the 1.5–5.19 Mb region on chromosome 1 included two QTLs for PC, J-*qPC1.1* (this study) Q-*qPC1.1* (Qin et al., 2009), and four QTLs for AACs. Two major QTLs, *qAAC6.1* and *qAAC7.1* were novel, only detected in this study.

## Discussion

Highly nutritional rice may contain high PC with desirable AA constitutions. Detecting the QTL which controls PC and AAC of the rice grain would provide a basis for improving nutritional qualities of the rice grain. In this study, 155 RILs and 1,327 markers were used to identify QTL for PC and AAC in grains. The genetic map showed an average of a 280.9 kb distance between the markers and an average of 0.93 markers within a 1-cM distance. Most of the studies for AAC and PC employed mapping populations from the crosses between *indica* and *japonica*. In the inter-subspecific crosses of rice, hybrid barriers, such as segregation distortion, hybrid sterility, and hybrid breakdown occur (Harushima et al., 2001; Jiang et al., 2008; Chin et al., 2011; Reflinur et al., 2014; Kim et al., 2017). Due to these reasons, several linkage gaps are shown in some chromosomal regions. Similarly, several large gaps existed in the genetic map constructed in this study. Lacking the proper number of markers or loci in the regions, the identification of some important QTLs might have not yet been identified and characterized.

A significant positive correlation among the majority of AAs were found in M23/T887 RILs (Supplementary Figure S1). This result is consistent with a previous study using Dasanbyeo (*indica*)/TR22183 (*japonica*) RILs (Yoo, 2017). In addition, it has been reported that several AACs were controlled by a single QTL in previous studies (Wang et al., 2007; Lu et al., 2009; Yoo, 2017). Similar results for several AACs have been found in this study (Figure 2). A total of 11 and 10 AACs, for instance, were affected simultaneously by *qAAC6.1* and *qAAC7.1*, respectively. These results suggest that these QTLs are related to the upstream regulator of AA biosynthesis, which would simultaneously affect contents of several AAs.

To validate the effects of *qAAC7.1*, M23-*qAAC7.1*^T887^, possessing homozygous *qAAC7.1*^T887^ in the M23 genetic background, was selected (Figure 5A). This line showed improved contents of most AACs, except Thr, Pro, and Lys (Figure 5B). In addition to AACs affected by *qAAC7.1* in the primary QTL analysis, PC and other AACs, including Met, His, Lys, and Gly, were also significantly higher than those of both parents, indicating that *qAAC7.1*^T887^ could confer high AACs and PC consistently.

Transgressive segregations were observed for all AAC and PC traits, appearing values higher or lower than both parents (Figure 1). This result indicates that both parental lines have alleles that contribute to AACs and PC at several loci, and progenies that able to have higher contents by containing favorable alleles derived from each other donor parent.

The favorable alleles of QTLs for AAC and PC were evenly detected in both *japonica* and *indica* parents. For example, among three positive alleles of QTLs for His contents, *qAAC1.1* and *qAAC1.6* (total PVE = 11%) were derived from M23, while *qAAC1.2* (PVE = 13.7%) originated from T887 (Supplementary Table S2). Similarly, for *qPC1.1*^T888^, *qPC1.2*^*M*23^, and *qPC7.1*^*M*23^, positive alleles for PC were also derived from each other parent. These results imply that pyramiding favorable alleles by inter-subspecies cross were an effective strategy to develop highly nutritional rice, improving AAC and PC of the grain. The QTLs for AACs and PC identified in this study would be useful in developing high nutritional rice.

## Data Availability Statement

The GBS data is available at NCBI under the accession numbers PRJNA601019 and PRJNA264250. Raw genotyping data of each RIL is available in a Supplementary Data Sheet 1.

## Author Contributions

SJ, C-KK, and JC designed the experiments. SJ, J-HH, YL, and N-HS conducted the experiments and collected the data. SJ, C-KK, YK, and JC analyzed the data. J-HH and N-HS collected the phenotypic data. SJ and JC wrote the manuscript. All authors read and approved the manuscript.

## Conflict of Interest

The authors declare that the research was conducted in the absence of any commercial or financial relationships that could be construed as a potential conflict of interest. The handling Editor declared a past co-authorship with one of the authors, JC.
