# Supplementary material for: Mapping and Validation of QTLs for the Amino Acid and Total Protein Content in Brown Rice
Source: Front Genet. 2020 Mar 17;11:240. doi: 10.3389/fgene.2020.00240 (PMC7089939; doi:10.3389/fgene.2020.00240)
Supplement: Supplementary file 1 [file Presentation_1.pdf]

Supplementary Material

Supplementary Table 1. Primers used in this study

Target QTL	Primer	Forward sequence	Reverse sequence	Size (bp)	
				T887	M23
qAAC7.1	id4638739	TCACATGTCTTCACTTTTCAACCA	TAACTCACAAACTCCTCCTGCC	173	142
	id4734109	TCGTTTGACTGACATAAACCTAGGA	TGACGTACTGTTCACCGTGATT	153	130
	id4819110	TGACATGTCCAAAAGGTTTAGAATT	GCAAACTAGAATTAACTTGCTCGA	180	153
	id4906614	TGAGCAACTAATGTTTTTCCGGT	CAAACACATACCTCAACGCTCA	155	173
	id5056584	AATTTTGCATGCTGGTGAAATTG	AGAAAAGTCTATTCATCCCTCGAGG	126	111
	id5200843	CTTGATGAAAAGGGGATCGGAGG	CAAATTCTGGCATCCTCCCTTTG	131	107
	id5227020	ACTTATTAGCGGGCCCGTAATT	AGTACGAACAAACTGCATGCAC	166	134
qAAC6.1	id3428419	GAGCTCGAGCCAAATCAATGAA	GCGATATGGGATATGTTGACCC	114	97
	id4203043	TGACTGTGTAATTTAGCCCACCA	ACCTAAGTATCGGAGGTACCAA	150	125

Supplementary Table 1. Primers used in this study

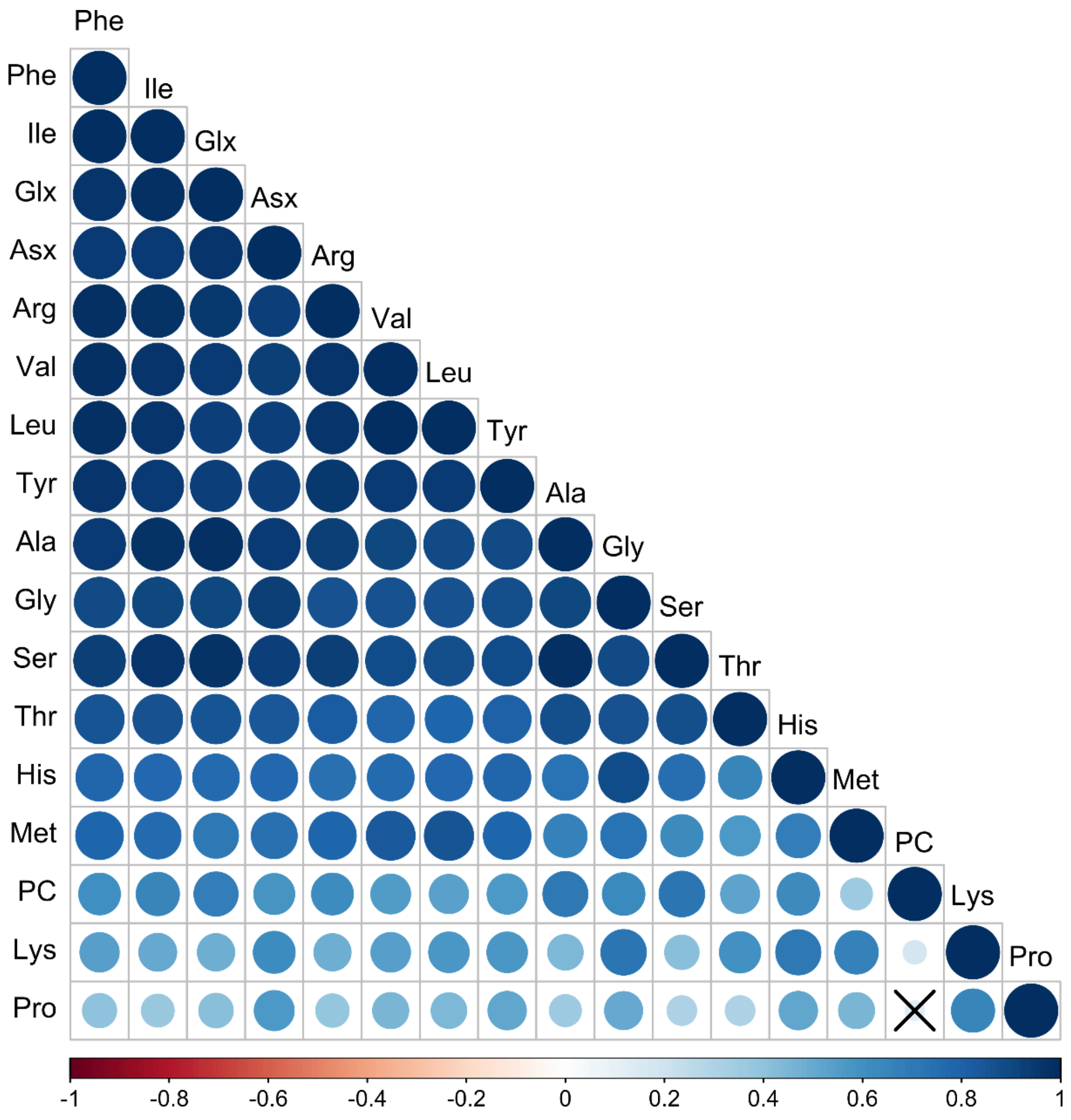

Supplementary Figure 1. The correlation matrix plot of AACs and PC. The correlation matrix plot represents Pearson's coefficient. Blue and red dots represent positive and negative correlation, respectively. The larger size and thicker color of dots represent the larger value of correlation. X marks insignificant coefficients ($p < 0.05$).

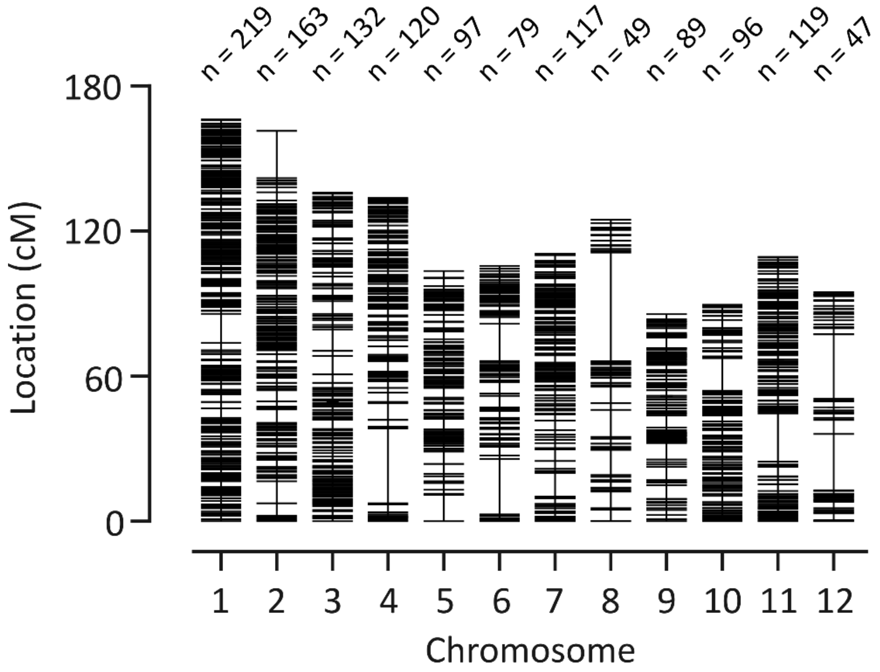

Supplementary Figure 2. Genetic map constructed using 155 M23/T887 RILs. A total of 1,327 bin markers were used to construct the genetic map. *n* represents the number of markers on each chromosome.

Supplementary Table 2. PC and AAC QTLs identified in T887 / M23 RILs

Chr[a]	QTL	Trait	QTL position (Mb)	LOD[b]	PVE (%)	Add	positive allele
1	*qAAC1.1*	His	3.21	5.82	6.25	0.1153	M23
	qAAC1.2	His	4.40	11.73	13.71	-0.1687	T887
	qAAC1.3	Ser	4.67	22.96	8.34	-0.5241	T887
	qPC1.1	PC	4.67	5.71	11.7	-0.0005	T887
	qAAC1.4	Ser	5.18	16.41	5.12	0.4175	M23
	qAAC1.5	Ser	37.41	7.01	1.89	0.2554	M23
	qAAC1.6	His	39.20	4.46	4.76	0.1006	M23
	qPC1.2	PC	39.20	8.68	18.12	0.0006	M23
2	*qAAC2.1*	Ser	4.14	3.6	0.94	-0.1739	T887
	qAAC2.2	Ser	18.11	51.7	29.47	-0.9769	T887
3	*qAAC3.1*	Ala	4.58	2.57	5.49	-0.1832	T887
	qAAC3.2	Gly	5.18	2.75	3.64	-0.1322	T887
6	*qAAC6.1*	Ala	3.59	6	13.75	0.3013	M23
		Arg		3.57	9.81	0.4136	M23
		Asx		4.02	11.09	0.4340	M23
		Glx		4.32	11.41	1.0224	M23
		Gly		5.56	7.84	0.1987	M23
		Ile		3.89	10.17	0.3929	M23
		Leu		4.09	11.68	0.2083	M23
		Met		16.9	10.81	0.3381	M23
		Phe		4.14	11.32	0.2751	M23
		Tyr		3.56	10.37	0.1216	M23
		Val		4.32	12.35	0.2956	M23
	qAAC6.2	Met	4.21	9.3	5.23	-0.2479	T887
7	*qAAC7.1*	Arg	4.94	4.29	12.15	-0.4402	T887
		Ile		5.84	15.92	-0.4699	T887
		Leu		2.96	8.44	-0.1693	T887
		Phe		4.29	11.92	-0.2698	T887
		Ala	5.11	6.95	15.66	-0.3072	T887
		Asx		5.17	14.1	-0.4677	T887
		Glx		6.08	16.28	-1.1690	T887
		Ser		11.34	3.24	-0.3248	T887
		Tyr		2.86	8.17	-0.1033	T887
		Val		3.08	8.56	-0.2353	T887
	qPC7.1	PC	5.72	4.63	9.42	-0.0004	T887
	qAAC7.2	Thr	5.72	5.2	15.33	-0.2440	T887
	qAAC7.3	Gly	13.16	3.27	4.46	-0.1426	T887
8	*qAAC8.1*	Gly	22.70	2.66	16.3	-0.2727	T887
	qAAC8.2	Lys	26.45	3.08	9.31	-0.1754	T887

[a]Chromosome; [b]Logarith of odds for QTL peak; ,[c]Phenotypic variance explained by QTL; ,[d]Addtive effect of QTL.

Supplementary Table 2. PC and AAC QTLs identified in RILs

Supplementary Table 3. List of QTLs for PC and AAC in the present and previous studies

Chr[a]	Trait	QTL	Markers	Position[b] (Mbp)	Parents[c]	Reference of QTLs[d]
1	PC	*qGPC1.1*	Afx93237905-Afx93229368	0.6-1.11	AN	C
	PC	*qPC1.1*	RM10080-RM575	1.5-8.1	SN	Q
	His	*qAAC1.1*	2886294-3539052	2.9-3.5	MT	J
	His	*qAAC1.2*	4301442-4494576	4.3-4.5	MT	J
	Ser	*qAAC1.3*	4632918-4708475	4.6-4.7	MT	J
	PC	*qPC1.1*	4632918-4708475	4.6-4.7	MT	J
	Ser	*qAAC1.4*	5154287-5197935	5.15-5.19	MT	J
	Asp/Thr/Ser/Glu/Gly/Ala/Val/Ile/Leu/Phe/His/Arg/Pro/Total[e]	*qAa1*	RM493-RM562	12.3-14.6	ZD	Z
	PC	*qPr1*	RM493-RM562	12.3-14.6	ZD	Z
	PC	*qPC1*	YJInDel-34–YJInDel-536_2	26.2-29.4	YJ	K
	Ser	*qAAC1.5*	37112359-37711021	37.1-37.7	MT	J
	Eaa[f]/Total/Asp/Thr/Glu/Gly/Ala/Cys/Tyr/Pro	*qAA.1*	RM472-RM104	37.9-40.2	ZN	W
	His	*qAAC1.6*	39162572-39234399	39.16-39.23	MT	J
	PC	*qPC1.2*	39162572-39234399	39.16-39.23	MT	J
2	Ser	*qAAC2.1*	4065908-4220624	4.1-4.2	MT	J
	PC	*qSGPC2.1*	Affx93260438-Affx93236905	5.17-6.17	AN	C
	PC	*qPC-2*	RM5897-RM6247	5.8-6.7	CN2	L
	Val/Ile/Leu/His/Phe	*qAA.2*	RM322-RM521	7.4-10.8	ZN	W
	PC	*qPC2*	YJInDel-61–YJInDel-67	8.0-13.5	YJ	K
	His	*qAA.2*	RM324-RM301	11.4-12.2	ZN	W
	Ser	*qAAC2.2*	18092703-18131695	18.09-18.13	MT	J
3	Ala	*qAAC3.1*	4542873-4614153	4.5-4.6	MT	J
	Gly	*qAAC3.2*	5140419-5225129	5.1-5.2	MT	J
	PC	*qPC-3*	RM251-RM282	10.0-12.4	XM	Y2
	PC	*qPC3*	YJInDel-128–YJInDel-130	22.8-24.7	YJ	K
	Tyr	*qAA.3*	RM520-RM468	30.9-32.7	ZN	W
	Ala/Val/Leu/Ile/Phe	*qAla3*	id3015453-id3016090	32.5-33.7	DT	Y3
4	Thr/Gly/His/Arg	*qAA.4*	RM348-RM131	32.7-34.4	ZN	W
6	PC	*qPC-6*	RM190-RZ516	1.8-2.6	XM	Y2
	Ala/Arg/Asx/Glx/Gly/Ile/Leu/Met/Phe/Tyr/Val	*qAAC6.1*	3515361-3658340	3.51-3.65	MT	J
	Met	*qAAC6.2*	4192641-4230522	4.19-4.23	MT	J
	PC	*qPC6*	YJInDel-207–YJInDel-208	5.2-7.9	YJ	K
7	Arg/Ile/Leu/Phe/Ala/Asx/Glx/Ser/Tyr/Val	*qAAC7.1*	4856196-5206110	4.9-5.2	MT	J
	Pro/Gly/Met/Arg	*qAA.7*	RM125-RM214	5.5-12.8	ZN	W
	Thr	*qAAC7.2*	5627191-5803738	5.6-5.8	MT	J
	PC	*qPC7.1*	5627191-5803738	5.6-5.8	MT	J
	Gly	*qAAC7.3*	11635687-14689865	11.6-14.7	MT	J
	PC	*qPr7*	RM445-RM418	17.5-18.1	ZD	Z
	PC	*qSGPC7.1*	Affx93225742-Affx93256949	22.2-22.3	AN	C
	PC	*qPC.1*	R1245-RM234	24.3-25.5	ZM	T
8	PC	qPro-8	RM506-RM1235	0.13-1.21	CN1	Y1
	PC	*qPC8*	YJInDel-306–YJInDel-320	3.0-4.1	YJ	K
	PC	*qPC8*	YJInDel-321–YJInDel-324	5.1-5.7	YJ	K
	PC	*qPC8*	YJInDel-324–YJInDel-340	5.7-8.6	YJ	K
	Tyr	*qAA.8*	RM137-RM556	13.9-22.3	ZN	W
	Gly	*qAAC8.1*	19156876-26250116	19.2-26.3	MT	J
	Lys	*qAAC8.2*	26398897-26510920	26.4-26.5	MT	J
	Cys	*qAA.8*	RM447-RM458	26.5-27.4	ZN	W
9	PC	qPro-9	RM219-RM23914	7.2-7.9	CN1	Y1
	Asp/Thr/Ser/Gly/Val/Ile/Phe/Lys/Total	*qAa9*	RM328-RM107	19.7-20.1	ZD	Z

10	Cys/Leu/Ile/Phe	*qAA.10*	RM467-RM271	13.5-16.7	ZN	W
	PC	*qPC-10*	RM184-RM3229B	16.4-16.7	XM	Y2
11	PC	*qPC11.1*	11027-RM287	16.5-17.2	SN	Q
	PC	*qPC11.1*	RM287-RM26755	17.2-17.6	SN	Q
12	PC	*qPC12*	YJInDel-510–YJInDel-515	23.6-25.4	YJ	K

[a]Chr. chromosome

[b]Physical positions of the markers were determined using IRGSP 1.0 Nipponbare reference genome (Sakai et al., 2013)

[c]Parents of mapping population. MT, Milyang23/Tong88-7; AN, ARC10075/Naveen; CN1, Cheongcheong/Nagdong; CN2, Chuan7/Nanyanghan; DT, Dasanbyeo/TR22183; SN, Samgang/Nagdong; XM, Xieqingzao B/Milyang46; YJ, Yukihikari/Joiku462; ZD, Zhenshan97B/Delong208; ZM, Zhenshan97/Minghui63; ZN, Zhenshan97/Nanyangzhan

[d]Reference of QTLs. J, this study; C, Chattopadhyay et al., 2019; K, Kinoshita et al., 2017; L, Lou et al., 2009; Q, Qin et al., 2009; T, Tan et al., 2001; W, Wang et al., 2007; Y1, Yun et al., 2014; Y2, Yu et al., 2009; Y3, Yoo, 2017; Z, Zhong et al., 2011.

[e]total, content of all amino acid; [f]EAA, the total amount of essential amino acid.

Supplementary Table 3. List of QTLs for PC and AAC in the present and previous studies
